# A Mini Review on CO_2_ Capture and Separation Using Nanocellulose-Based Scaffolds

**DOI:** 10.3390/polym18161971

**Published:** 2026-08-13

**Authors:** Priyanka Sharma

**Affiliations:** Department of Chemical and Paper Engineering, Western Michigan University, Kalamazoo, MI 49008, USA; priyanka.sharma@wmich.edu; Tel.: +1-(269)-276-3506

**Keywords:** nanocellulose, CO_2_ capture, CO_2_ separation, gas-separation, sustainability

## Abstract

Atmospheric carbon dioxide (CO_2_) has reached an unprecedented 430 ppm, warming the planet by 50% compared with pre-industrial times and prompting a search for a quick and effective solution to control CO_2_ emissions. As a robust, renewable, biodegradable, and sustainable material, nanocellulose can serve as a strong support for many active molecules. Nanocellulose, whether in suspension, aerogel, or membrane form, is not sufficient for efficient CO_2_ capture and separation; hence, active molecules, such as silanes, amines, zeolites, and metal–organic frameworks (MOFs), are introduced via chemical modification, such as grafting, or via physical mixing as fillers or additives to make nanocellulose effective for CO_2_ capture and separation. Introducing amine or silane molecules into nanocellulose has proven to be an effective strategy for achieving a satisfactory CO_2_ absorption capacity exceeding 6 mmol/g. Nanocellulose membranes, when fabricated with MOFs or zeolites and used as a coating with polyvinyl alcohol (PVA) to create a thin-film composite membrane (TFC), can achieve CO_2_ permeance of more than 600 GPU for CO_2_ separation from flue gas, with CO_2_/N_2_ selectivity close to 40. This review provides an overview of nanocellulose-based CO_2_ capture and separation materials developed over the last 10 years, along with the related challenges that must be overcome to meet current performance and demand. To facilitate readability, the author has provided a brief introduction to the origin, performance, and scale-up developments of nanocellulose at the start of this review.

## 1. Introduction

Atmospheric carbon dioxide (CO_2_), the main greenhouse gas, has reached an unprecedented level of 430 ppm, warming the planet by 50% compared with pre-industrial times [1]. It contributes significantly to global warming. For example, the disappearance of the icebergs is evidence of the adverse impacts of global warming and warns us to adopt sustainable, viable measures to control greenhouse gas emissions [2]. CO_2_ is a chemically stable molecule due to the covalent bonds between carbon and oxygen atoms, and the presence of a double bond further stabilizes it, thereby preventing further transformation and reactivity under atmospheric conditions [3]. CO_2_ is not only the primary and critical carbon sink on Earth but also a valuable component in several industrial processes [4]. Hence, if CO_2_ from point sources and from direct air is captured and stored for reuse to create additional value, this strategy could help mitigate CO_2_ emissions and promote sustainability and environmental health. Most CO_2_ emissions result from human activities, particularly the combustion of fossil fuels such as coal, oil, and natural gas. For example, approximately 33% of CO_2_ emissions come from energy and electricity generation, about 14% from transportation, and about 12% from manufacturing and construction [5]. To control CO_2_ emissions from these sources or sectors, two primary approaches, Point-source Capture and Direct Air Capture (DAC), are used at the sites. Point-source capture involves capturing CO_2_ directly at the facility before it is released into the atmosphere. Hence, it is more applicable when CO_2_ concentrations are higher at emission sites, such as in large industries like cement, steel, chemical, and fertilizer. For high-emission sites, high-volume scrubbing materials such as zeolites, metal oxides, and amines are often used. While DAC involves extracting CO_2_ from the atmosphere, it requires significant energy to capture the very dilute CO_2_ in large volumes of air to achieve zero emissions. To facilitate the DAC process, molecules such as amines, aminosilanes, and specialized MOFs that form strong chemical interactions with atmospheric CO_2_ are used. These molecules are applied as liquid scrubbers, membranes, or solid adsorbents. Liquid scrubbers mostly operate via chemical absorption, where interactions between the scrubbers’ functional groups and CO_2_ affect overall performance; membranes operate via the size-exclusion principle, where mostly pore size and porosity affect overall performance; and solid adsorbents operate primarily on surface area and functionality. The performance of all these materials is highly dependent on temperature, pressure, and humidity. For example, some specialized zeolites such as Zeolite 13X, Zeolite 5A, and Lithium-exchanged X Zeolite (LiX) [6] are used in the Pressure Swing Adsorption (PSA) technique to separate and purify gases from a mixed stream at high pressure under non-cryogenic conditions, while the pressure drop is used to release the trapped gases. This technique is an efficient way to capture and separate CO_2_ but is associated with high energy use. Therefore, current technologies face challenges related to operation, high energy consumption, corrosion, and environmental unfriendliness. Hence, there is an ongoing need for research and development to identify sustainable, efficient materials that overcome these challenges while maintaining and enhancing performance.

Among these, nanocellulose has emerged as a promising material because of its combined sustainability and exceptional structural and physicochemical properties, which enable further chemical functionalization and structural transformation into form a gel, a porous material, and a robust support, all suitable for CO_2_ capture [7,8]. This review concisely covers the origins of different nanocellulose types from cellulose, the properties of nanocellulose that support CO_2_ capture and separation, and the progress over the last 10 years in nanocellulose-based CO_2_ capture and separation materials, including their properties and performance, the related challenges, and future perspectives for further advancing these materials. The SciFinder database was used with the keywords ‘nanocellulose in CO_2_ adsorption and separation’ and ‘CO_2_ capture and separation using nanocellulose’.

## 2. Cellulose and Nanocellulose

**Figure 1:**
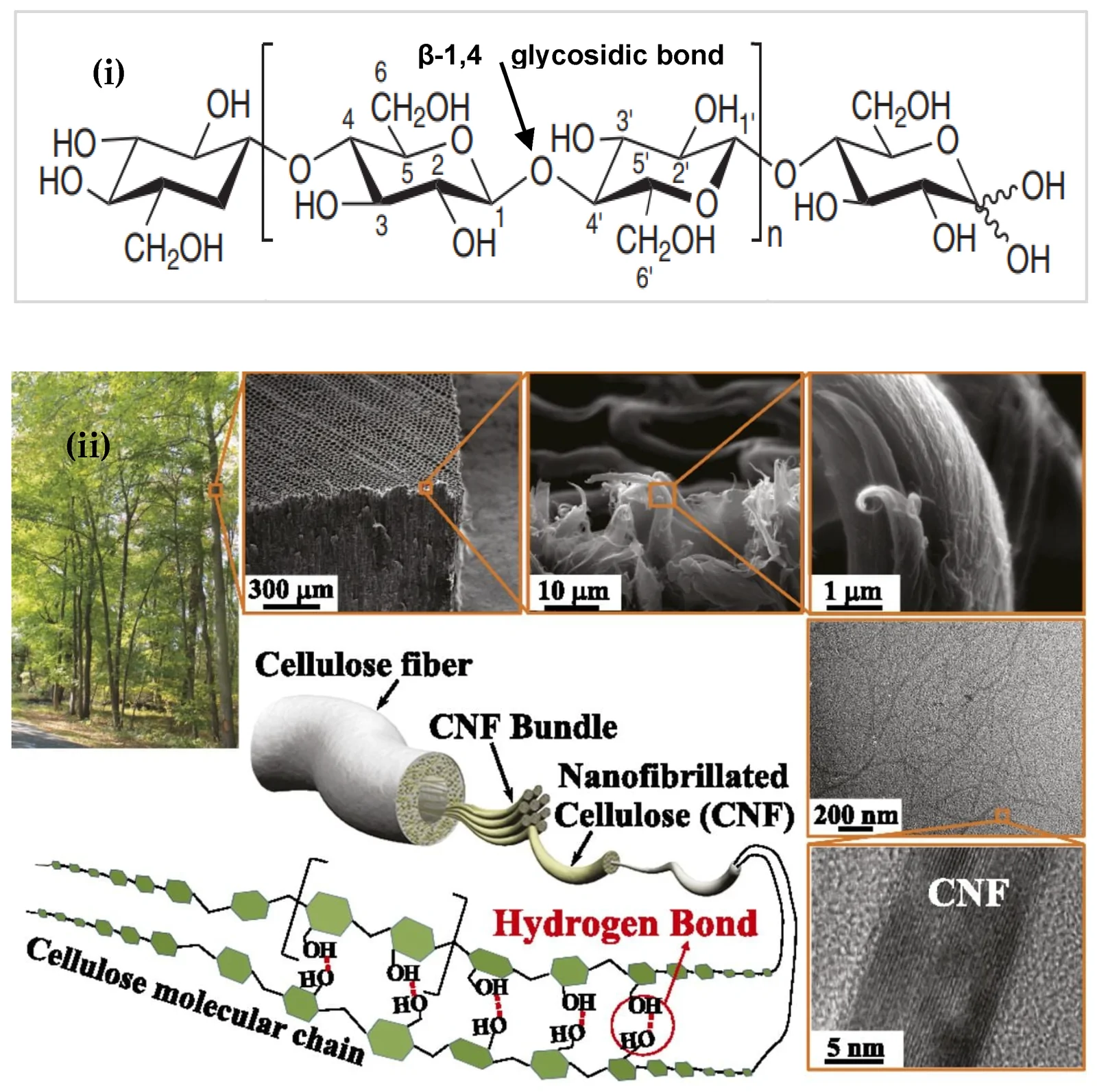
(**i**) Cellulose chain chemical structure. (**ii**) Wood cellulose fiber hierarchical structure [11].

Cellulose is a semicrystalline polysaccharide found as a major component of plant cell walls (hardwood, softwood, grasses, and agricultural residues), bacterial cell walls, some tunicates, and algae. From a chemical point of view, it is a long chain of anhydroglucose units, which are bound together with β-1,4- glycosidic bonds (Figure 1(i)). Due to the abundance of hydroxyl groups, i.e., two secondary hydroxyl and one primary hydroxyl group, in each unit, these lead to cellulose chains with strong hydrogen bonding. Both intermolecular and intramolecular hydrogen bonding are present, and the hydroxyl groups play a significant role in forming them [9]. A simple hierarchical structure of plant cellulose fiber is presented in Figure 1(ii). The extracted cellulose from the plant cell wall usually has a diameter of 20–100 µm, and each cellulose fiber is composed of a bundle of cellulose microfibrils, each with a width > 15 nm. Each bundle of microfibrils comprises microfibrils with a diameter of 3–4 nm, assembled via intermolecular and intramolecular hydrogen bonding [10]. All the hydrogen bonds from hydroxyl groups are engaged in strong hydrogen bonding, as shown in Figure S1 of the Supplementary Information; therefore, defibrillating the microfibrils into nanofibrils requires immense energy. The microfibril structure consists of both crystalline and non-crystalline (amorphous) regions, representing the tightly bound and loosely bound (easily accessible) regions, respectively. The existence of these two forms plays a significant role in the evolution of different nanoforms of cellulose fibers.

**Figure 2:**
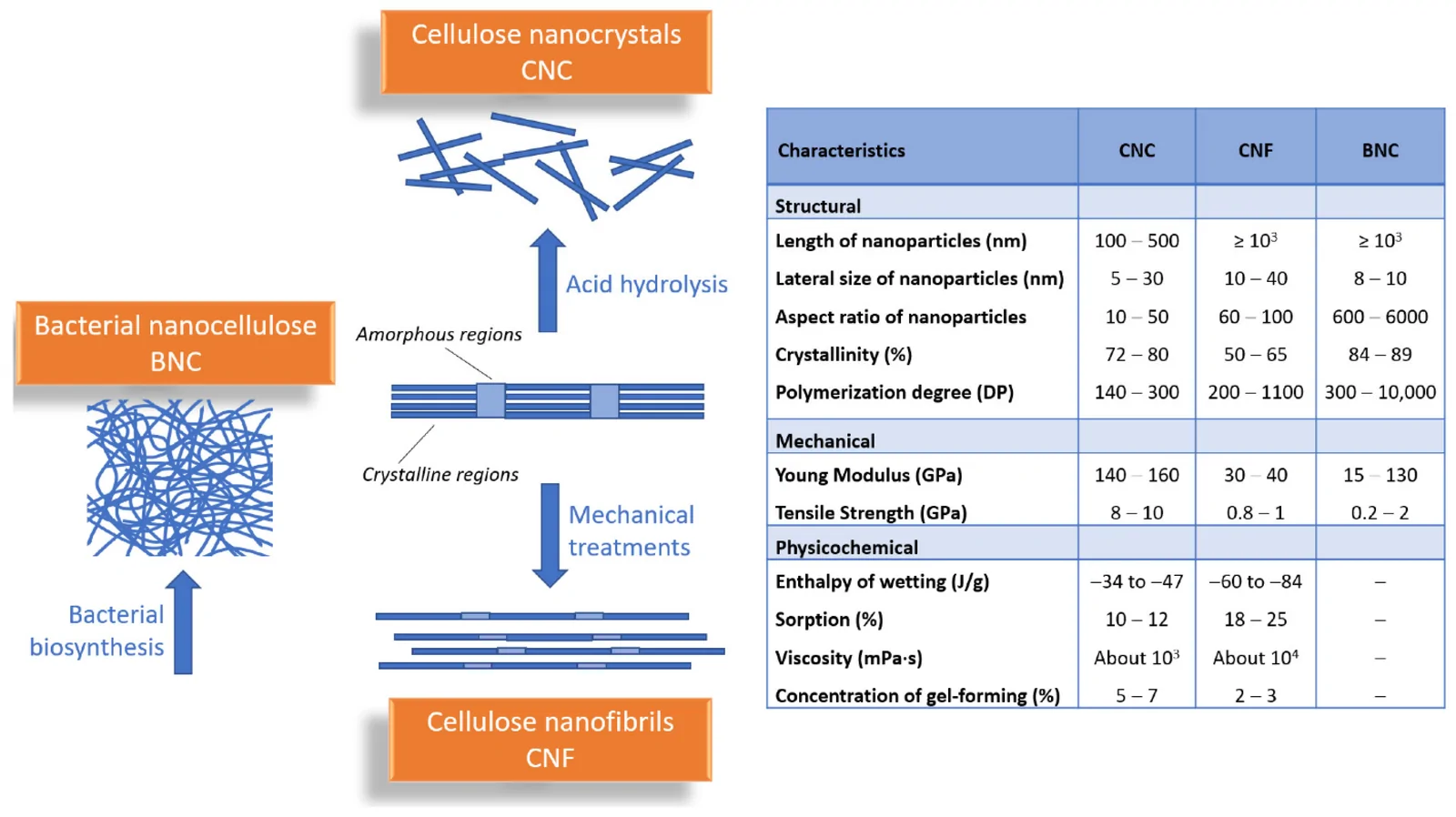
Preparation scheme and properties of cellulose nanocrystals (CNCs), cellulose nanofibers (CNFs), and bacterial nanocellulose (BNCs) [13].

Cellulose nanoforms, also called nanocellulose, are characterized by high aspect ratio, high surface area, and high tensile strength (>220 GPa), even stronger than Kevlar [12]. The preparation scheme and structural, mechanical, and physicochemical properties of different nanocellulose types (cellulose nanocrystals (CNCs), cellulose nanofibers (CNFs), and bacterial nanocellulose (BNCs)) are provided in Figure 2. In brief, CNCs are fabricated using acid hydrolysis, CNFs are synthesized using mechanical treatment, and BNCs are synthesized by bacteria, as presented in Figure 2. A detailed description is provided in Section S1 and Figure S2 in the Supplementary Information.

## 3. Nanocellulose in CO_2_ Capture and Separation

**Figure 3:**
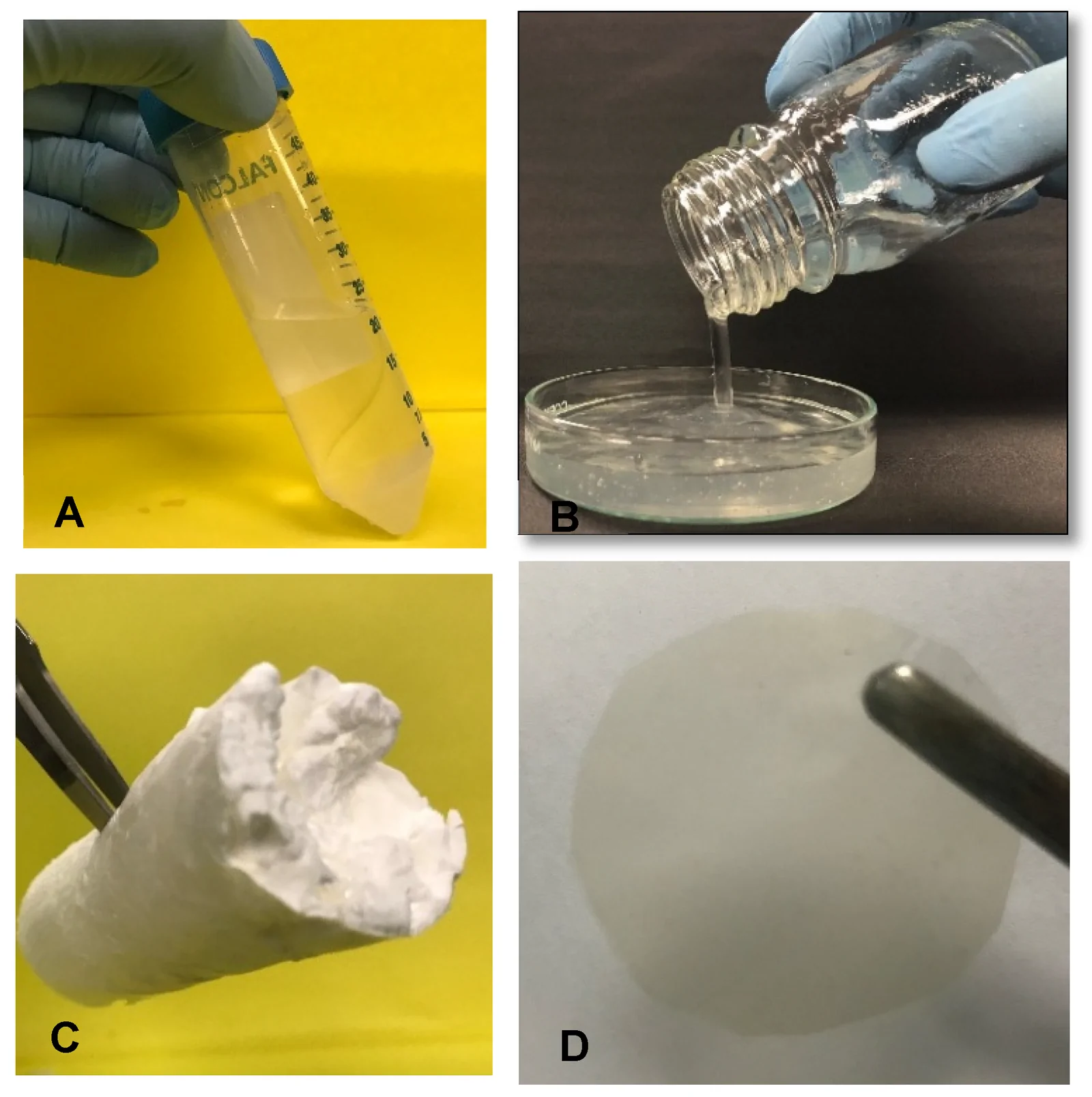
(**A**) Nitro-oxidized cellulose nanofibers suspension (0.05 wt%), (**B**) nitro-oxidized cellulose nanofibers gel (0.25 wt%), (**C**) nitro-oxidized cellulose nanofibers aerogel, (**D**) nitro-oxidized cellulose nanofibers membrane prepared from solvent casting.

Cellulose fibers are limited to solid forms, with limited dispersion and solubility, whereas once converted to nanocellulose, they appear in suspension and gels [14], (as shown in Figure 3A,B), which exhibit greater dispersion and miscibility for use in composites, additives, strengthening materials, and fillers across a wide range of applications [15]. Once nanocellulose is dried using advanced lyophilization techniques, it can be converted into high-surface-area and highly porous aerogels (as shown in Figure 3C). The specific porosity and pore size in aerogel can lead to efficient CO_2_ capture. However, if the nanocellulose is oven-dried, the nanoscale fiber structure collapses and appears as a hard sheet form [16]. However, if microfiltration with solvent evaporation is used, it can appear as a low-porosity membrane (as shown in Figure 3D) [17]. A low-porosity membrane can lead to better selectivity in gas separation applications. Along with this, the nanocellulose suspension shows gelation, or an increase in viscosity, with increasing concentration or upon interaction with metal ions [18,19]. Achieving such features in virgin cellulose fibers is challenging; therefore, nanocellulose has expanded the application of traditional cellulose fibers to more advanced applications in sensors, energy storage, catalysis, and paper packaging, etc., and is playing a vital role across fields, from medicine to packaging, to enhance sustainability and promote the circular economy in the current era of an advancing world [20]. This review provides concise information on the structural and physicochemical transformation of nanocellulose to make it efficient for CO_2_ capture and separation applications. In this review, to refer to all structural nanocellulose forms (suspension, gel, aerogel, and membrane), the term nanocellulose scaffold is used.

Mechanisms involved in CO_2_ capture by nanocellulose include:

(1) Physical Interaction: This includes the interaction between the nanocellulose surface and CO_2_ through van der Waals interactions and pore filling in aerogels or nanocellulose porous structures. In general, physical adsorption is reversible, has a low adsorption enthalpy (typically 10–14 kJ/mol), and exhibits a low adsorption capacity. Unmodified nanocellulose, which is abundant in hydroxyl groups, absorbs CO_2_ through physisorption, and due to a lack of strong CO_2_-reactive sites, it typically shows very low adsorption below 1 mmol/g under ambient conditions [21].

(2) Chemical Interaction: Chemical interaction includes building covalent bonds between CO_2_ and relevant functional groups (primary and secondary amines, polyethyleneimine (PEI), amino silanes). Chemical interaction shows high selectivity, strong adsorption, and excellent low-pressure performance. It typically shows a stronger adsorption energy of 40–100 kJ/mol [21].

Chemical Reaction 1 describes the reactivity of amine-containing nanocellulose with CO_2_. The reaction generally forms a carbamate (RNHCOO^−^) and a protonated primary amine (RNH_3_+), thereby breaking down the CO_2_ molecule.

CO_2_ + RNH_2_ ---> RNHCOO^−^ + RNH_3_^+^

Therefore, higher amine loadings often increase chemisorption, leading to greater CO_2_ capture. Here, a lone pair on the amine nitrogen attacks the electrophilic carbon of CO_2_.

Chemical Reaction 2 describes the reactivity of alkaline-modified nanocellulose with CO_2_ to form the carbonate. The preparation of alkaline-modified nanocellulose is an exothermic process, performed with a high concentration of 4–20% alkali.

CO_2_+ NaOH ---> NaHCO_3_

In the next section, the different structural and chemical modifications of nanocellulose for CO_2_ capture and separation applications are discussed.

### 3.1. Nanocellulose-Based Composite for CO_2_ Capture and Separation

Nanocellulose forms such as CNCs, CNFs, and BNCs differ in their aspect ratio, crystallinity, and functionality; therefore, their mechanical and physicochemical properties (as shown in Figure 2), including mechanical strength, viscosity, and interaction behavior with other molecules, vary. That is, when used in capture and separation applications, either as such or as a composite in the form of an aerogel, membrane, or coating, as explained in Section 3.1 (i–v), the ultimate properties of the final scaffold could differ. For example, CNCs and BNCs are fibrous in nature; hence, they can be entangled to make a flexible network. In such cases, this may reduce free-volume efficiency for gas diffusion, yielding no significant improvement in CO_2_ transport performance. For example, when CNCs were blended with Pebax 1657 to prepare the hybrid membrane. The CNCs, which are rod-like and highly crystalline, exhibited enhanced CO_2_ permeability and selectivity. The addition of 5 wt% CNCs to Pebax 1657 resulted in 42% and 18% increases in CO_2_ permeability and CO_2_/N_2_ selectivity, respectively, to 305.7 Barrer and 41.6, respectively. The rigid CNCs maintain structural stability and prevent membrane swelling or collapse under higher humidity and in a high-pressure gas stream [22,23]. Hence, the use of CNCs for applications under non-ambient conditions is suitable and can help achieve appropriate performance and efficiency. However, fibrous CNFs and BNCs could be useful for achieving more defined and robust porous structures, such as aerogels and foams, and for surface modification with CO_2_-active sites for CO_2_ adsorption purposes [24]. Fibrous CNFs and BNCs can be used to achieve a high surface area and mesoporous pore sizes in aerogels, thereby improving the diffusion for efficient CO_2_ capture and separation [25]. Functional groups on nanocellulose and their interactions with active molecules again impact CO_2_ capture and separation ability. For example, succinate groups were grafted onto CNFs, thereby enhancing CO_2_ capture with Pebax. The addition of 0.5 wt% modified CNFs to Pebax enhanced the tensile strength and separation performance, yielding CO_2_ permeability of 263.8 Barrer and CO_2_/CH_4_ selectivity of 19.9 at 4 bar and 30 °C. The use of succinate-modified CNCs with Pebax has probably enhanced CO_2_ diffusion in Pebax [26].

**Figure 4:**
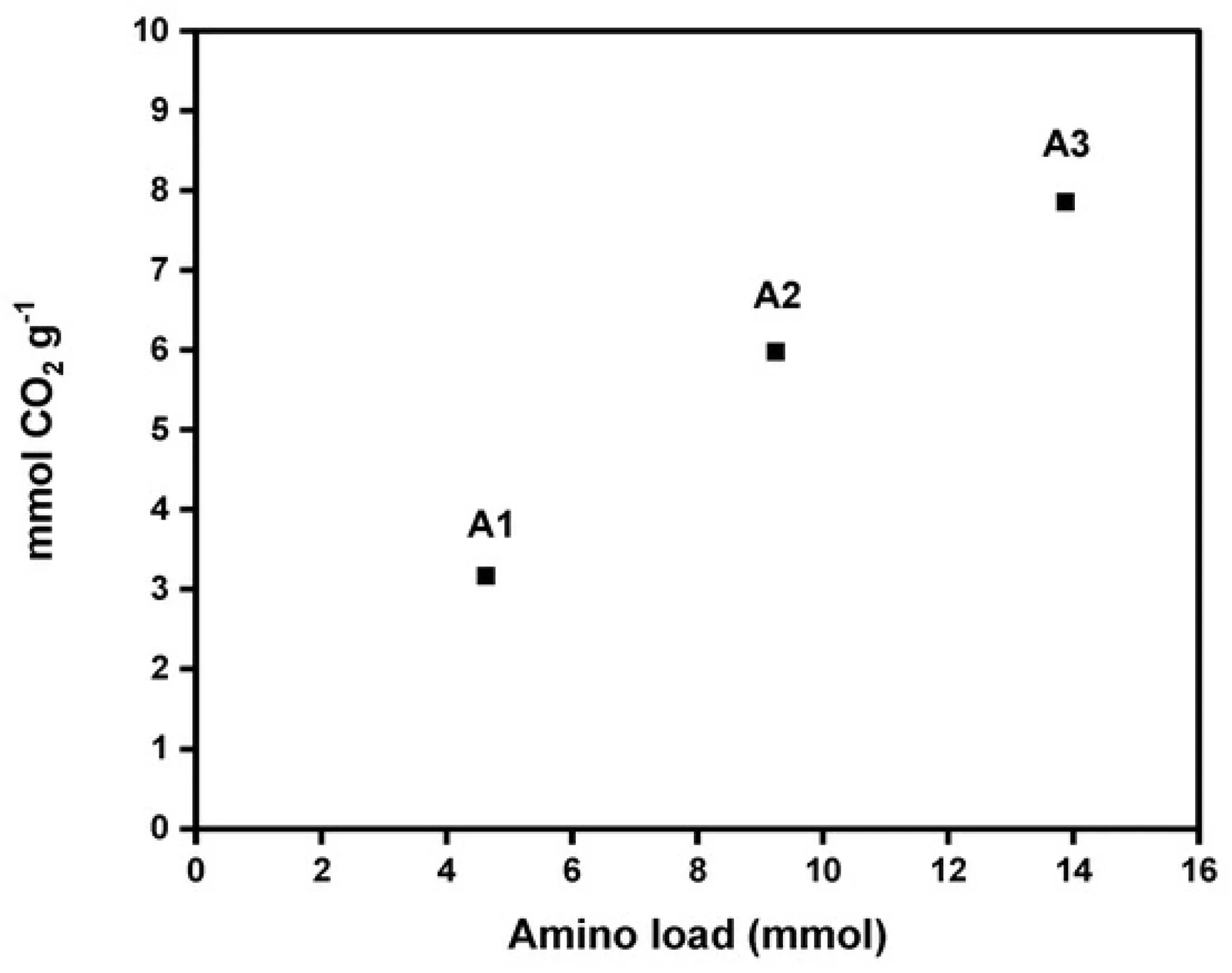
Amino group load effect on Silylation of Nanocellulose aerogel using N-[3-(trimethoxysilyl) propyl] ethylenediamine (DAMO)-modified nanocellulose aerogels calculated by the pressure decay technique [31].

(i) Aerogels and Membranes: Nanocellulose aerogels are used as CO_2_ adsorbents. These aerogels were prepared by drying highly concentrated nanocellulose fibers via lyophilization, followed by further treatment with a solvent or via crosslinking. For example, Li et al. [27] used a Ca^2+^-crosslinked CNFs membrane to adsorb CO_2_, achieving an efficiency of 3.4 mmol/g after 30 days of operation. However, in another study, the CNFs aerogel, when impregnated with cellulose acetate, showed an absorbance of 1.14 mmol/g at 101 kPa and 273 K [28], whereas after carbonization and impregnation with clay, it showed an absorbance of 12.14 mmol/g. The CO_2_ absorption of the aerogel was primarily controlled by pore size, and based on the above literature, filler impregnation effectively improved the aerogel’s efficiency.

(ii) Amines or Amides at Nanocellulose: Other major nanocellulose substrates used for CO_2_ removal included the incorporation of amine molecules, either through grafting or mixing, into nanocellulose scaffolds. Amine is basic in nature, and the lone pair of electrons on the nitrogen of the amine makes it highly reactive towards acidic CO_2_ molecules. This allows the amine moieties to selectively remove CO_2_ and release it on heating. In this regard, nanocellulose (CNFs or CNCs) was used as a base material for the grafting of polyamines, dimethoxysilane, polyvinylamine, and L-arginine to remove CO_2_. Among these, when polyallylamine (PAM) and functionalized CNFs were coated on a polyvinylidene fluoride (PVDF) membrane, it showed a 652 barrer, with CO_2_/N_2_ selectivity of 41.3 [29]. Polyether amide block, such as Pebax MH1657, is also used with CNCs as a filler in polyurethane composites, where CNCs, when used with Pebax 1657, showed a CO_2_ permeability of 305.7 barrer and a CO_2_/N_2_ selectivity of 41.6 [30]. Such a polyether amide bond interacts with a CO_2_ molecule via Lewis’s acid-base interactions, in which the ether oxygen (highly electronegative) strongly interacts with the electropositive central carbon atom. Valdebenito et al. [31]. discussed the controlled silylation of nanocellulose aerogel using N-[3-(trimethoxysilyl)propyl]ethylenediamine (DAMO). The study showed that chemisorption was the predominant mechanism for CO_2_ capture, and the CO_2_ adsorption capacity increased with higher amino group loading (4.62, 9.24, and 13.87 mmol of DAMO). In Figure 4, A1, A2, and A3 indicate nanocellulose aerogel with 4.62, 9.24, and 13.87 mmol of DAMO amino loading, respectively. At 298 K and 4 bar, CO_2_ adsorption capacity increased proportionally with the amino group concentration, reaching values of 3.17, 5.98, and 7.86 mmol of CO_2_ g^−1^ polymer, respectively. This silane-modified aerogel maintained around 95% CO_2_ desorption at ambient temperature for 20 adsorption/desorption cycles [31]. 

(iii) MOFs and Zeolites on nanocellulose: MOFs are a highly porous scaffold, where metal ions are surrounded by organic frameworks. This specific arrangement of metal ions with organic molecules makes it highly porous, so 1 g of MOF can cover the area of one football field. Therefore, their use in environmental remediation continues to rise. Nanocellulose, being strong and mechanically robust, is used as a supporting material for MOFs. In one study, an MOF-CNFs film was coated onto filter paper with nano- and macroscale fiber morphologies, demonstrating high CO_2_/CH_4_ selectivity (over 120 for CO_2_) at high gas flux [32]. In another study, a CNF gel containing ZIF-90 was ion-exchanged and then coated onto filter paper. The carboxy functionality on CNFs enabled strong interactions with MOFs, resulting in a dense network that limited CO_2_ leakage. This membrane exhibited the CO_2_/CH_4_ selectivity of 123 [32]. Zhang et al. have demonstrated a hybrid membrane comprising CNFs and a zirconium-based MOF (UiO-66) with a pore size of ~6 Å for CO_2_ separation, and reported a CO_2_ permeance of 139 Barrer [33]. In another study, Jia et al. made a hybrid of CNFs and MOF ZIF-8 on a nylon substrate. The membrane showed a CO_2_ permeability of 550 Barrer and excellent CO_2_/N_2_ selectivity of 45 and CO_2_/CH_4_ selectivity of 36 [34]. The membranes’ enhanced performance resulted from strong crosslinking between Zn^2+^ in the MOF and carboxylate groups in the CNFs. Recently, HKUST-1 MOF was incorporated into nanocellulose, and this membrane showed a further enhancement in CO_2_/CH_4_ selectivity to 123 [34]. 

(iv) DES/Ionic Liquid (IL) at nanocellulose: A hybrid membrane comprising CNFs and the ionic liquid (IL) 1-ethyl-3-methylimidazolium acetate ([Emim][OAc]) was prepared with controllable pores, together exhibiting exceptional separation properties. In this study, IL loadings of 20, 35, and 50 wt% relative to the membrane solids content were tested; however, increasing IL content enhances the water uptake capacity of the CNF film. At optimal IL loadings and humidity levels, the membrane exhibited a CO_2_ permeability of ~330 Barrer and an excellent CO_2_/N_2_ selectivity of 330 [35,36]. This study emphasizes the importance of the network architecture of partially swollen CNF fibrils in selectively permeating CO_2_ through enhanced diffusive pathways. Humidity strongly affects the selectivity of nanocellulose, and this behavior can be precisely tuned to control gas transport properties. A deep eutectic solvent (DES) containing choline chloride and ZnCl_2_ has also been used with nanocellulose, showing a CO_2_ permeance of 330 Barrer and a CO_2_/N_2_ selectivity of 43.6 [37].

(v) Nanocellulose coating on membrane: Nanofibers of carboxymethylated cellulose were coated on PVA and sterically hindered polyallylamine (SHPAM) TFC membrane. This hybrid membrane showed excellent performance, with CO_2_ permeance up to 652 GPU and a CO_2_/N_2_ selectivity of 41.3 [29]. In another study, incorporating CNF into the polyvinylamine-dense membrane improved its mechanical properties. Notably, the amine groups in polyvinylamine facilitated CO_2_ transport; however, the membrane was sensitive to humidity, as the CNFs swelled upon exposure to moisture [38]. The polyvinylamine membrane composed of 30% CNFs demonstrated CO_2_ permeability of 187 Barrer, CO_2_/N_2_ selectivity of 100, and CO_2_/CH_4_ selectivity of 22 at a relative humidity of ~80%. In another study, CNCs, phosphorylated CNCs, and CNFs were blended with PVA and coated on a polysulphone membrane, and the relative CO_2_ permeance and selectivity were reported. PVA/CNC nanocomposite membranes demonstrated an excellent CO_2_ permeance of 128 GPU and an increased CO_2_/N_2_ separation factor of 39 compared with PVA composite membranes, which showed a permeance of 105.5 GPU and a separation factor of 36. In this study, the various concentrations of CNCs from 0.5 to 4 wt% were blended with 2 wt% of PVA solution at pH 9 before coating on the polysulfone membrane. The increase in humidity from 30 to 100% and the CO_2_/CH_4_ selectivity of the PVA nanocomposite membrane containing 1.5 wt% CNCs increased from 6 to 39. When CNCs and PLA were coated onto poly(p-phenylene oxide) (PPO) hollow fibers, a maximum CO_2_ permeance of 672 GPU and a CO_2_/N_2_ selectivity of 43.6 were reported. This membrane also exhibited excellent long-term stability for up to 1 year. The presence of nanocellulose at the polymer interface creates a gas transport channel that enhances CO_2_ permeation. The studies also indicate that high surface charge and small size enhance gas-separation properties. Due to the swelling behavior of nanocellulose, the gas selectivity of the nanocellulose hybrid membrane can vary. For example, a membrane composed of polyvinylamine and CNFs, loaded with CNFs at 30–70 wt%, was tested for humid gas permeation; the membrane exhibited different selectivity. The membrane with CNFs and 70% polyvinylamine showed selectivity of 135 for CO_2_/CH_4_ and 218 for CO_2_/N_2_ at 60% relative humidity, and a maximum permeability of 187 Barrer at 80% relative humidity [39]. Table 1 summarizes the properties of nanocellulose-based CO_2_ adsorbents. Table 2 presents a summary of the properties of nanocellulose-based CO_2_ capture and separation membranes.

### 3.2. Parameters Affecting Efficiency of Nanocellulose-Based Materials for CO_2_ Capture and Separation

Parameters such as pressure, humidity, and temperature affect the performance of nanocellulose scaffolds for CO_2_ capture.

1. Pressure: Increasing system pressure increases interactions between functional groups on the cellulose surface and CO_2_, thereby enhancing efficiency [59]. For example, the impact of feed pressure (5, 10, 15 bar) was investigated on a CNFs-coated polyvinylamine (PVAm) nanocomposite membrane. It was observed that CO_2_ permeance decreased by a factor of 1.8 as pressure increased from 5 to 15 bar for the PVAm membrane containing 1 wt% CNC [39].

2. Humidity: The increase in humidity enhances CO_2_ adsorption by facilitating CO_2_ dissolution in polar water molecules and stabilizing the amine-based sorbent. For example, CNFs-PEI aerogel with a PEI loading of 44 wt% showed an increase in CO_2_ capture capacity from 1.5 to 2.22 mmol/g as humidity increased from 40 to 80% [40]. Similarly, a hybrid film containing L-arginine and carboxymethyl cellulose, when tested for CO_2_ permeability at 35 °C across humidity levels from 70 to 98%, showed higher permeability at 94% humidity (220 Barrer) than at 70% humidity (29 Barrer). Similarly, CNFs and lupamine aerogel showed an increase in permeability from 135 to 187 Barrer for CO_2_/N_2_ separation as humidity increased from 60 to 80% [23].

3. Temperature: Increasing the temperature decreases CO_2_ capture (adsorption) due to reduced bond strength between CO_2_ and amino groups [60]. The overall efficiency of the nanocellulose-based CO_2_ adsorbent can be defined by the adsorption capacity, regeneration efficiency, and gas diffusion rate. The rise in temperature increases molecular randomness, decreasing the overall uptake of CO_2_ molecules by the nanocellulose scaffold, while also facilitating the release of captured or adsorbed CO_2_ molecules. Hence, the alteration in temperature could have a mixed overall impact; therefore, these parameters should be well studied before their technical applications.

### 3.3. Nanocellulose Large Scale Production

The global nanocellulose market has been growing due to sustainability demands, the unique and versatile properties of nanocellulose, and its environmental benefits. While cellulose is renewable, sustainable, biodegradable, and sourced from plant fibers or agricultural residues, the fibrillation process to transform it into nanoscale dimensions is chemical- and energy-intensive. Hence, studies show that for highly chemical-intensive processes, the CO_2_ emissions per unit mass are temporarily higher than those of conventional materials such as Portland cement or polylactic acid [61]. In such cases, a milder treatment, such as a base treatment, combined with mechanical fibrillation, can be used to produce ligno-nanocellulose that is less energy- and chemical-intensive and can be applied to make it more efficient with a low carbon footprint. Still, the market value of nanocellulose is increasing, valued at USD 351.5 million in 2022 and expected to reach USD 1517.5 million by 2030, with a compound annual growth rate (CAGR) of 20.1% from 2023 to 2030 [62]. Many companies have successfully produced nanocellulose, as presented in Table 3. Among these production facilities, CelluForce, located in Canada, is one of the earliest commercial-scale CNC production facility, which utilize the acid hydrolysis process to transform wood pulp into CNCs. While FiberLean Technologies has sites in India, France, and the UK, it is leading the MFCs scale-up through the mechanical grinding of pulp fibers. Other companies, such as GranBio Technologies, use their own proprietary methods to produce nanocellulose from lignocellulosic biomass. The University of Maine- Nanocellulose Center has expanded its scope by its own production of CNFs, but also by providing the selling platforms for start-ups and new companies. It indicates that, in the coming years, the facile application of nanocellulose to meet sustainability and environmental demands could be achievable across most sectors. The bulk use of nanocellulose as a supporting scaffold with CO_2_-reactive sites could be achieved if further performance enhancements are made by increasing the structural, physical, and mechanical properties of nanocellulose scaffolds, thereby also improving the scaffolds’ regenerative ability.

## 4. Conclusions and Future Perspectives

Nanocellulose, a sustainable alternative with a low carbon footprint, offers several advantages, including low cost, biodegradability, enhanced recyclability, a robust surface, tunable functionality, and the ability to form porous structures. The presence of inherent hydrogen bonds due to hydroxyl groups helps establish strong bonding among the fibrils themselves and with incoming supporting molecules; therefore, the strength of the nanocellulose and cellulose composite can be maintained when it is dry. However, the presence of moisture (evident in the environment) causes the nanocellulose to lose its integrity and structure, thereby reducing its efficiency. This review discusses how the inherent properties of nanocellulose are utilized to fabricate adsorbents, membranes, and films, either alone or in hybrid forms, to achieve effective CO_2_ absorption and removal with good selectivity. Given that CO_2_ is an acidic oxide, its reaction with a base such as an amine can lead to neutralization and the trapping of CO_2_ molecules. Hence, this review discusses reported nanocellulose aerogels, films, and membranes bearing amine moieties that bind either through chemical reactions or ionic interactions. Examples of the use of nanocellulose as a support material for highly porous MOFs, Mxenes, and zeolites are also included in this review. Hence, this review provides significant protocols for nanocellulose, including its properties, commercial availability, and its use as a membrane, film, or aerogel, either in single or hybrid forms, for CO_2_ capture.

**Future Perspective**: Although nanocellulose has played a critical role as a robust scaffold, either by itself or in a hybrid matrix, for capturing CO_2_, and has enhanced the recyclability of main CO_2_ capture molecules such as amines, MOFs, silanes, zeolites, and MXenes, there are still challenges that must be overcome to make it viable and scalable. For example, the hydrophilicity of the nanocellulose scaffold causes it to swell in high-humidity environments, thereby reducing the number of active sites. Therefore, research should focus on chemically modified nanocellulose with some hydrophobicity. This may help maintain performance and enhance the recyclability of the whole system. Another challenge is that most reported studies are conducted in controlled lab settings, which limits the technical application of such materials. Hence, future studies should also be tested under real-world conditions, such as varying temperature, humidity, and contaminants, to evaluate the actual performance of nanocellulose scaffolds. The cost analysis and life cycle assessment of nanocellulose scaffolds for CO_2_ capture and reuse are also limited; a detailed study could assess the viability and efficiency of this material for technical applications. Modifying nanocellulose into multifunctional nanocellulose composites, optimizing hierarchical pore architecture, developing moisture-resistant adsorbents, integrating nanocellulose with MOFs and porous organic polymers, employing machine learning for material design, evaluating long-term performance under industrial flue gas conditions, and advancing DAC based on biopolymeric (nanocellulose and nanocellulose hybrid) materials could help improve and better test the feasibility and efficiency of nanocellulose-based CO_2_ capture and separation scaffolds.

## Figures and Tables

**Table 1:** Summary of the properties of nanocellulose scaffolds and their CO_2_ capture performance. Adsorption performance for nanocellulose-based CO_2_ adsorbents is depicted as CO_2_ adsorption capacity (mmol/g).

Substrate	CO_2_ Adsorption Capacity (mmol/g)	Test Conditions	Ref.
CNFs-DAMO	7.86	At 298 K 4 bar	[31]
CNFs-PEI aerogel	2.22	80% relative humidity 25 °C, 1 atm	[40]
CNCs silylation with triethoxysilyl propyl-3-pentanedinitrile carbamate	5.54	Ambient temperature	[41]
CNFs aerogel silylation with AEAPMDS	1.91	Ambient temperature	[42]
CNFs aerogel modified with APMDS	2.26	25 °C, humid CO_2_ capture capacity increased with humidity	[43]
CNFs aerogel modified with Triethoxysilylpropyl-3-pentanyldinitrile carbamate hydrolyzed and polymerized with Na_2_SiO_3_	2.20	25 °C	[44]
Ca^2+^ crosslinked CNFs membrane	3.4	Ambient condition	[27]
CNFs aerogel impregnated with cellulose acetate	1.14	101 kPa, 273 K	[28]
CNCs modified with PAMAM	3.54	25–45 °C	[45]
CNFs modified with PNIPAm	6.23	25 °C, Humid conditions (Thermorespons-ive)	[46]
CNF aerogel modified with APMDS	1.01	Ambient condition	[47]
CNC aerogel modified with AEAPMDS	2.63	3 bars	[48]

**Table 2:** Summary of the properties of nanocellulose-based CO_2_ capture and separation membranes. CO_2_ permeability indicates gas transport capability and is reported in Barrer units. CO_2_ permeance indicates thin-film membrane performance and is reported in Gas Permeance Units (GPU). CO_2_/N_2_ selectivity indicates flue gas separation. CO_2_/CH_4_ selectivity indicates natural gas sweetening.

Substrate	CO_2_ Permeability or Permeance	Selectivity Test Conditions	Ref.
PVA/PAM/functionalized CNFs coated on a PVDF membrane	652 GPU	CO_2_/N_2_ = 41.3 100% RH, RT	[29]
CNFs coating on polyvinylamine	187 Barrer	CO_2_/N_2_ = 100 CO_2_/CH_4_ = 22 5–15 bar	[49]
CNCs-Pebax 1657	305.7 Barrer	CO_2_/N_2_ = 41.6 2 bar, 30 °C, 100% RH	[23]
CNFs-Lupamin	218 barrer	CO_2_/CH_4_ = 135 CO_2_/N_2_ = 218 60% RH	[39]
L-arginine-carboxymethyl nanofibers film	220 Barrer 94% RH	CO_2_/N_2_ = 185 94% RH, 35 °C	[38]
Polyethylene oxide-PEBAX MH1657, polyurethane, and nanocellulose composite membrane	>90 Barrer	CO_2_/N_2_ = 70 4 bar, 25 °C	[50]
**MOFs/Zeolite on** **Nanocellulose**			
Regenerated cellulose-ZIF-90	2970 GPU	CO_2_/CH_4_ = 123 0.2 MPa, 25 °C	[32]
Nanocellulose on HKUST MOF		CO_2_/N_2_ = 298 1 bar, 25 °C	[51]
CNC-PLA onto PPO hollow fiber membrane	672 GPU	CO_2_/N_2_ = 43.6 3 bar, 25 °C	[52]
CNF-UiO-66	139 Barrer	CO_2_/N_2_ = 43.6 1 bar, 25 °C	[33]
CNF-ZIF-8	550 Barrer	CO_2_/N_2_ = 45.5 CO_2_/CH_4_ = 36.2 1 bar, 25 °C	[34]
Nanocellulose-NaY-zeolite	266.7 Barrer	CO_2_/N_2_ = 92.4 0.1 MPa, 25 °C	[53]
**Nanocellulose with DES/Ionic Liquid**			
Nanocellulose with Choline chloride and ZnCl_2_	155 Barrer	CO_2_/N_2_ = 43.6 CO_2_/N_2_ = 48.4 Ambient conditions	[37]
CNF-[Emim][OAc]	330 Barrer	CO_2_/N_2_ = 370 1 bar, 30 °C	[54]
**Nanocellulose coating on the membrane**			
PVA/CNCs on PPO	672 GPU	CO_2_/N_2_ = 436 0.2 bar, 25 °C	[55]
Phosphorylated cellulose nanocrystals/PVA-L-arginine coating in facilitated transport membranes	25,772 Barrer	CO_2_/CH_4_ = 10 90% RH and different feed pressures (0.8–1 bar) Increase in Phosphorylated CNC enhanced CO_2_ permeability but reduced selectivity.	[56]
**Others**			
Mxene-Nanocellulose-DES	496.7 Barrer	CO_2_/N_2_ = 78.8	[57]
Mxene filler in nanocellulose scaffold	156.7 Barrer	CO_2_/N_2_ = 42.6 CO_2_/CH_4_ = 47.8 The entire testing process was carried out at steady state, and the transmembrane pressure was maintained at a nearly constant level between 0.1 and 0.5 MPa.	[58]

**Table 3:** Major nanocellulose producers and their production capacities.

Company and Location	Production Capacity	Methodology	Reference
CelluForce Montreal, Canada	300 dried metric tons/year	Acid hydrolysis to produce CNC	[63]
FiberLean Technologies Saint Austell, Cornwall, UK	2000 dry metric tons per annum of fibril capacity	Mechanical treatment of pulp fibers to produce Microfibrillated cellulose (MFC)	[64]
GranBio Technologies São Paulo, Brazil, Thomaston/Marietta, GA, USA	½ ton/day Facility, Georgia, USA	Use BioPlus technology to extract nanocellulose from various biomass	[65]
CelluComp Ltd. Renville, MN, USA, Edinburgh, UK	400 tons annually	Extract Nanocellulose from root vegetables nanocellulose to produce Curran^®^	[66]
Nippon Paper Industries Tokyo, Japan	30 tons annually in Ishinomaki, Japan	Extract nanocellulose from wood pulp	[67]
Sappi Johannesburg, South Africa	Not mentioned	Extract CNC branded as ‘Valida’ from wood-derived cellulose	[68]
University of Maine Orono, ME, USA	1 ton per day	Utilize mechanical processes to produce nanocellulose	[69]
SWFT Labs, LLC East Setauket, NY, USA	Not mentioned	Extract nitro-oxidized cellulose nanofibers from non-woody biomass	[70]

## Data Availability

No new data were created or analyzed in this study. Data sharing is not applicable to this article.

## Supplementary Materials

The following supporting information can be downloaded at: https://www.mdpi.com/article/10.3390/polym18161971/s1, Figure S1: Hydrogen bonding in native cellulose (cellulose I); Figure S2: Pictorial presentation of the mechanism of the transformation of cellulose into various cellulose nanoforms.

## Abbreviations

MOFs	Metal-organic frameworks
TFC	Thin Film Composite Membrane
PVA	Polyvinyl alcohol
CO_2_	Carbon dioxide
PAM	Polyallylamine
PVDF	Polyvinylidene fluoride
DAMO	N-[3-(trimethoxysilyl)propyl]ethylenediamine
[Emim][OAc]	1-ethyl-3-methylimidazolium acetate
DES	Deep eutectic solvent
SHPAM	Sterically hindered polyallylamine
PEI	Polyethylenimine
AEAPMDS	*N*-(2-aminoethyl)-3-aminopropyl methyldimethoxysilane
APMDS	3-aminopropylmethyldietoxysilane
PAMAM	Poly(amidoamine)
PNIPAm	Poly(N-isopropylacrylamide)
APMDS	N-(2-aminoethyl)-3- aminopropyl methyldimeth oxysilane
AEAPMDS	N-(2-aminoethyl) (3-amino-propyl) methyldimethoxyansile
PLA	Poly lactic acid
PPO	Poly(p-phenylene oxide)
PEI	Polyethyleneimine
DAC	Direct Air Capture
PSA	Pressure Swing Adsorption
CNCs	Cellulose nanocrystals
CNFs	Cellulose nanofibers
BNCs	Bacterial nanocellulose
IL	Ionic Liquid
Na_2_SiO_3_	Sodium silicate
