# Supplementary material for: A Mini Review on CO2 Capture and Separation Using Nanocellulose-Based Scaffolds
Source: Polymers (Basel). 2026 Aug 13;18(16):1971. doi: 10.3390/polym18161971 (PMC13516921; doi:10.3390/polym18161971)
Supplement: Supplementary file 1 [file polymers-18-01971-s001.zip › polymers-4394093-supplementary.pdf]

A Mini Review on CO_2 Capture and Separation Using Nanocellulose-based Scaffolds

Supplementary Information

Priyanka Sharma*

Department of Chemical and Paper Engineering, Western Michigan University, Kalamazoo, MI 49008, USA.

Corresponding Authors:

PRS: Tel: +1-(269) 276-3506; E-mail: priyanka.sharma@wmich.edu

S1. Cellulose Fiber Nanoforms

Cellulose fibers are limited to solid forms, with limited dispersion and solubility, whereas once converted to nanocellulose, they appear in suspension and gels,[17] which exhibit greater dispersion and miscibility for use in composites, additives, strengthening materials, and fillers across a wide range of applications. Like native cellulose, these are biopolymer, plant-derived, abundant, renewable, and environmentally friendly options. Hence, nanocellulose has expanded the application of traditional cellulose fibers to more advanced applications in membranes, sensors, energy storage, catalysis, and paper packaging, etc., and is playing a vital role across fields from medicine to packaging, to enhance sustainability and promote the circular economy in the current era of an advancing world. A large number of review articles have been published on nanocellulose chemistry, modification, structure–property relationships, characterization methods, composite and membrane preparation, applications, and life cycle assessment.

S.1.1. Brief Introduction on Cellulose Nanoforms

Cellulose nanoforms, or nanocellulose, are nanodimensioned cellulose fibers that retain the chemistry of cellulose fibers while offering enhanced properties. These nanocellulose can be extracted from different biomass sources. **Figure S2** depicts the mechanism of transforming cellulose fibrils into various nanoforms, including CNCs, CNFs, CMFs, MFCs, and spherical cellulose nanoparticles (SCNPs). The first cellulose nanoform studied was CNCs. CNCs were produced by acid treatment of cellulose, primarily inducing hydrolysis, which assisted in removing all loosely bound domains, i.e., amorphous regions. For example, sulfuric acid hydrolysis generates cellulose CNCs with sulfate ester groups on their surface, which are stable in colloidal dispersions. CNCs can

also be prepared by other acid treatments, such as hydrochloric acid, hydrobromic acid, and phosphotungstic acid. However, these acid treatments do not cause any significant change in the functionality of cellulose fibers.

Second, the cellulose nanoform, as shown in **Figure S2**, is made of CNFs, which are generated by mechanical treatment or mechanical cum chemical treatment of cellulose fibers, generally producing CNFs that are of high aspect ratio and have high mechanical strength. Mechanical treatment involves cryo-crushing, high-pressure homogenization, ultrasonication, ultrafine grinding, steam explosion, aqueous counter-collision, high-energy bead milling, homogenization, microfluidization, and enzymatic hydrolysis. Chemical treatments used to generate CNFs are TEMPO oxidation, esterification, nitro-oxidation, and phosphorylation. The chemical treatment of cellulose fiber causes the pulping of fibers and the introduction of a charge on their surface. When further subjected to mechanical treatment, charge repulsion causes the separation of individual microfibrils, known as CNFs.

The other form of nanocellulose, which is natively produced by bacteria, is Bacterial Nanocellulose (BNCs). In recent years, bacterial cellulose, including tubular bacterial cellulose, has attracted significant attention as a biomaterial with great potential for artificial blood vessels, especially small vascular grafts. Properties such as high purity, high strength, non-toxicity, high adsorption capacity, biocompatibility, high degree of polymerization (DP 4000-10000), and high crystallinity (CrI > 60%) make BNCs an important biomedical product. Many gram-negative and gram-positive bacteria, such as *Acetobacter*, *Azotobacter*, *Rhizobium*, *Pseudomonas*, *Salmonella*, *Alcaligenes*, and *Sarcina ventriculi,* can produce BNCs. BNCs have a high aspect ratio, with diameters of

20-100 nm and lengths of several micrometers, and consist of various nanofiber network types. Thus, BNCs do not require any further treatment to convert to a nanofiber; they are designed by nature to be a nanofiber.

Cellulose microfibrils (CMFs) or MFCs are partially fibrillated cellulose that can be generated by mechanical treatments. Other forms, such as spherical nanocellulose (SCNP), may be produced by simultaneously removing crystalline regions via chemical treatment, as shown in **Figure S2**. Nanofilaments of cellulose have also been reported in the literature, but have not been extensively researched. Due to sustainability demands, matching nanocellulose properties to current material and engineering requirements significantly promotes their scale-up production, as presented in the next section.

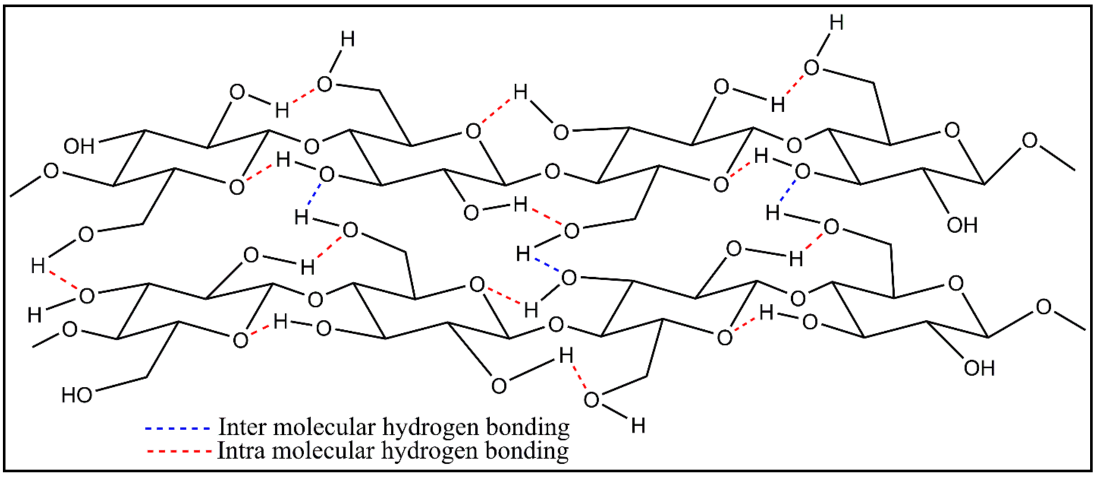

Figure S1. Hydrogen bonding in native cellulose (cellulose I).

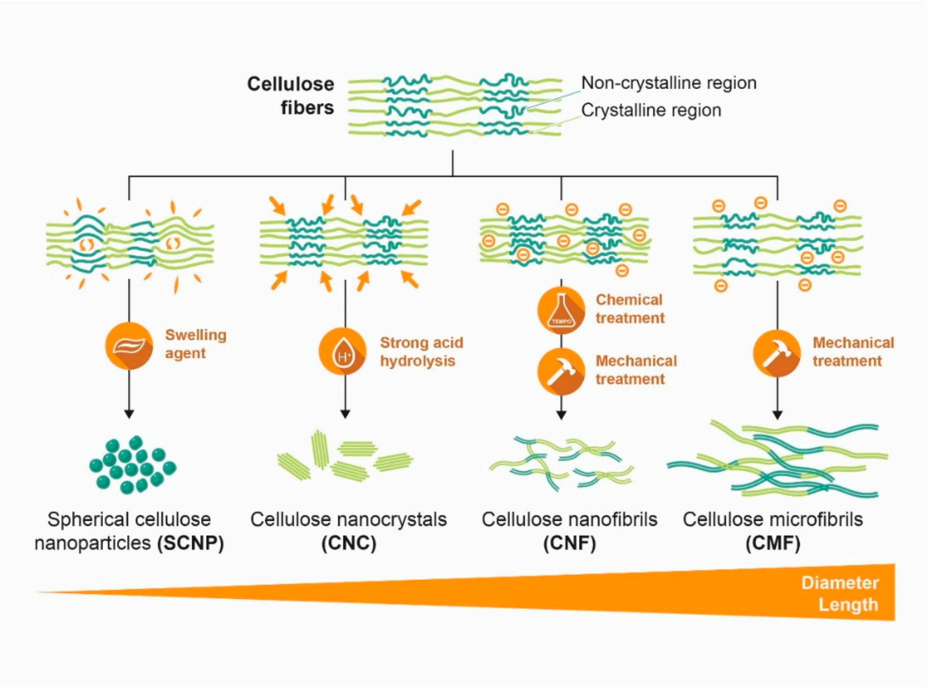

Figure S2. Pictorial presentation of the mechanism of the transformation of cellulose into various cellulose nanoforms.
